# Transport of Reactive Oxygen and Nitrogen Species across Aquaporin: A Molecular Level Picture

**DOI:** 10.1155/2019/2930504

**Published:** 2019-06-17

**Authors:** Maksudbek Yusupov, Jamoliddin Razzokov, Rodrigo M. Cordeiro, Annemie Bogaerts

**Affiliations:** ^1^Research Group PLASMANT, Department of Chemistry, University of Antwerp, Universiteitsplein 1, B-2610 Antwerp, Belgium; ^2^Centro de Ciências Naturais e Humanas, Universidade Federal do ABC, Avenida dos Estados 5001 CEP 09210-580 Santo André, SP, Brazil

## Abstract

Aquaporins (AQPs) are transmembrane proteins that conduct not only water molecules across the cell membrane but also other solutes, such as reactive oxygen and nitrogen species (RONS), produced (among others) by cold atmospheric plasma (CAP). These RONS may induce oxidative stress in the cell interior, which plays a role in cancer treatment. The underlying mechanisms of the transport of RONS across AQPs, however, still remain obscure. We apply molecular dynamics simulations to investigate the permeation of both hydrophilic (H_2_O_2_ and OH) and hydrophobic (NO_2_ and NO) RONS through AQP1. Our simulations show that these RONS can all penetrate across the pores of AQP1. The permeation free energy barrier of OH and NO is lower than that of H_2_O_2_ and NO_2_, indicating that these radicals may have easier access to the pore interior and interact with the amino acid residues of AQP1. We also study the effect of RONS-induced oxidation of both the phospholipids and AQP1 (i.e., sulfenylation of Cys_191_) on the transport of the above-mentioned RONS across AQP1. Both lipid and protein oxidation seem to slightly increase the free energy barrier for H_2_O_2_ and NO_2_ permeation, while for OH and NO, we do not observe a strong effect of oxidation. The simulation results help to gain insight in the underlying mechanisms of the noticeable rise of CAP-induced RONS in cancer cells, thereby improving our understanding on the role of AQPs in the selective anticancer capacity of CAP.

## 1. Introduction

In recent years, cold atmospheric plasma (CAP) application in cancer treatment has shown beneficial effects [1]. Experiments already evidenced that CAP may selectively eliminate cancer cells, leaving homologous normal cells less damaged [2–5]. This and other features of CAP, such as causing no pain in patients, no thermal and electrical damage, and low cost [6, 7], might give an advantage to CAP over traditional anticancer therapies.

CAP generates reactive oxygen and nitrogen species (ROS and RNS or RONS), e.g., H_2_O_2_, OH, NO, NO_2_, and O_3_. It is generally believed that the selective anticancer capacity of CAP is linked to the higher levels of RONS that are generated in cancer cells, while normal cells experience a relatively modest increase (if any) in RONS levels [4, 8–10]. The CAP-induced RONS diffuse across the cell membrane, causing nitrooxidative stress in the cell, thereby affecting the intracellular signaling pathways, and eventually leading to cell death [4, 11, 12]. However, the underlying mechanisms of the distinct increase of intracellular RONS are still not fully understood, although several explanations have already been proposed in the literatures [13–15].

One of the theories to explain the selective rise of intracellular RONS in cancer cells is based on the aquaporin (AQP) transmembrane proteins [13]. AQPs are mainly responsible for transporting water molecules across the membrane, but they can also conduct other permeants (such as H_2_O_2_, CO_2_, and NO) through their channels [16, 17]. AQPs are overexpressed in many cancer cells, including glioma, hemangioblastoma, lung adenocarcinoma, and laryngeal, colorectal, and ovarian cancer [18]. Recent experiments using AQP8 gene silencing in glioblastoma cells resulted in reduced toxicity of CAP-treated media towards these cancer cells [19]. Moreover, Miller et al. [20] found that the expression of AQP3 on colon adenocarcinoma HT29 cells, mammalian HEK 293 cells, and cervical cancer HeLa cells significantly enhanced the transport of H_2_O_2_ in these cells. Based on these observations, Yan et al. proposed that CAP-induced RONS may permeate into cancer cells considerably faster than into normal cells, through AQPs [13]. Hence, this difference in transmembrane permeation may lead to higher intracellular RONS concentrations, thereby resulting in cancer cell death [13, 19].

Following these considerations, we investigate here the transport of RONS across the AQP transmembrane protein, by means of molecular dynamics (MD) simulations. In addition, we consider the effect of CAP-induced oxidation on the transport of these RONS. Some simulation studies have already been performed, which are aimed at understanding the molecular level mechanisms of water conduction by AQPs (see, e.g., [21–25]). Besides, several studies were devoted to the permeation of different nonaqueous solutes across AQPs [26–30]. Cordeiro studied the permeation of ROS (specifically H_2_O_2_, HO_2_, and OH) across both mammalian and plant AQP models [31]. He found that all these species can permeate across the AQPs, with lower free energy barriers compared to those across the phospholipid bilayer (PLB) [31]. We recently investigated the transport of H_2_O_2_ across AQP1 [32]. Our simulations revealed that the permeability of H_2_O_2_ across AQP1 is at least two orders of magnitude higher than across the PLB, indicating that the delivery of H_2_O_2_ into the cell interior should be across AQP [32]. However, the overall mechanism of RONS permeation across AQP is still obscure. Moreover, there has been no systematic study on the effect of oxidation of both the PLB and AQP on the transport of RONS through AQP. This is of course highly relevant for a better understanding of the selective anticancer capacity of CAP, which produces various RONS and thus induces oxidation, as well as for other cancer therapies that are based on oxidation, such as chemotherapy, radiotherapy, and photodynamic therapy.

As the CAP-generated RONS reach the cell membrane, some are expected to enter the intracellular medium via AQPs or simple transmembrane diffusion. However, both the PLB and the AQPs are themselves targets to RONS-induced oxidation. It is conceivable that oxidation may change the properties of the PLB and the AQPs, which in turn generates a feedback loop on RONS permeability. In fact, experiments have shown that the AQP conductivity can be reversibly inhibited upon exposure to ROS [33–35]. It was proposed that direct oxidation of AQP or its surrounding phospholipids could trigger conformational changes that lead to channel closure. Recovery of the conductive state would then depend on cellular repair mechanisms. However, the existence of an oxidative gating mechanism remains to be demonstrated at the molecular level. It is also conceivable that, depending on the nature and extent of the oxidation process, the AQP permeability may be enhanced.

In this study, we carry out MD simulations to investigate the transport of RONS (specifically H_2_O_2_, OH, NO_2_, and NO) across the transmembrane protein AQP1 with molecular detail, as well as the effect of oxidation of both the PLB and AQP1 themselves. Specifically, we calculate the free energy profiles (FEPs) of RONS across (i) native AQP1, (ii) AQP1 surrounded by 50% oxidized phospholipids, and (iii) AQP1 containing oxidized Cys residues (see below).

## 2. Computational Details

We performed MD simulations of the permeation of H_2_O_2_, OH, NO_2_, and NO across AQP1. AQP1 is one of the members of the AQP family and widely expressed in different cancerous tissues, including breast cancer, colorectal cancer, astrocytoma, ovarian cancer, cervical cancer, and lung cancer [13]. We selected the above-mentioned species, to include two hydrophilic (H_2_O_2_ and OH) and two hydrophobic (NO_2_ and NO) representative RONS that are important in CAP treatment of cancer [11, 36].

**Figure 1:**
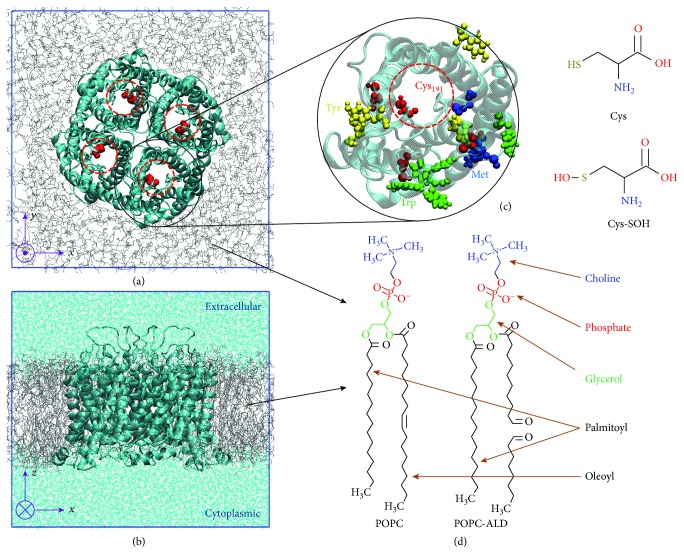
(a) Top and (b) side view of the NAT model system, i.e., the native AQP1 tetramer surrounded by a native POPC PLB (gray color) and covered with water layers on top and at the bottom (cyan color in (b)). The top and bottom water layers correspond to the extracellular and cytoplasmic aqueous regions. For clarity, the water layers are removed in (a). (c) Highlight of some of the AQP amino acid residues that are most reactive with OH radicals [40]. Cys_191_ is indicated with red dashed circles in (a) and (c). The chemical structures of Cys and its oxidized form Cys sulfenic acid (i.e., Cys-SOH) are given in (c), whereas the chemical structures of POPC and its oxidized form POPC-ALD are shown in (d). These lipids are used to construct the OXL model system, whereas Cys-SOH residues are used to build the OXP model system. Note that Cys_191_ is selected as the oxidation target in OXP because it can easily be oxidized and stays close to the ar/R selectivity region (see Figure 2).

Furthermore, we considered the AQP1 model in three possible states, called NAT, OXL, and OXP. NAT stands for native AQP1 (pdb id: 1J4N [37]) embedded in a fully hydrated native palmitoyl-oleoyl-phosphatidylcholine (POPC) bilayer (see Figures 1(a), 1(b), and 1(d)). We chose POPC as a model PLB because it is in the fluid state at the temperature applied in our simulations [38]. OXL also represents the native AQP1, but surrounded by an equimolar mixture of native and oxidized phospholipids. The oxidized phospholipid was considered as the product of oxidative acyl chain cleavage of POPC, which leads to lipid fragments bearing the aldehyde functional group (ALD) (see Figure 1(d)). This is indeed one of the major oxidation products [39]. It is formed from a ring closure and opening reaction of the intermediate lipid peroxide radical, which results in two aldehydes. Finally, OXP consists of oxidized AQP1 (obtained by modification of Cys_191_ of each monomer to Cys sulfenic acid, i.e., Cys-SOH, see Figures 1(a) and 1(c)), surrounded with native POPC PLB. Thus, NAT denotes the model system containing *native* AQP and PLB, whereas OXL and OXP represent the systems with either *oxidized lipids* or *oxidized protein*, respectively.

**Figure 2:**
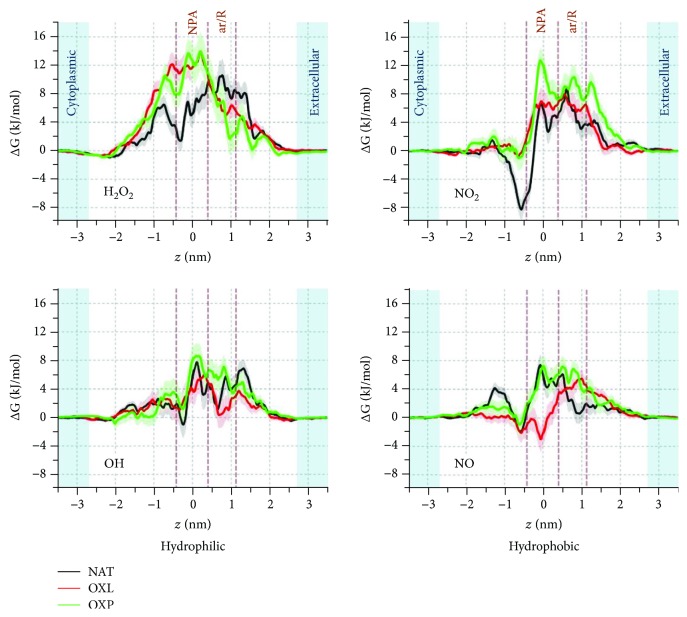
Average FEPs of the hydrophilic (H_2_O_2_ and OH) and hydrophobic (NO_2_ and NO) RONS across AQP1 pores of the native (NAT, black curve) and oxidized (OXL and OXP, red and green curves) model systems. The extracellular and cytoplasmic aqueous phases are shown in light blue color. The uncertainties in the profiles are represented in pale color. The NPA and ar/R regions are indicated by the brown dashed lines, and *z* = 0 nm is set to the NPA region. The same designation of the NPA and ar/R regions applies to the other similar figures below.

As is clear from Figure 1, AQP1 consists of four monomers each acting as an individual channel through which the transport of water and other solutes (e.g., H_2_O_2_ and NO) takes place. These monomers interact with each other through van der Waals forces and hence form the tetrameric complex. At the center of each monomer channel, two highly conserved Asn-Pro-Ala (NPA) motifs are located, which provide the selectivity against the permeation of H^+^ and other ions [31, 41]. Moreover, near the extracellular part of each channel, there is a constriction region, the so-called aromatic/Arg (ar/R), which also contributes to the selectivity. Note that in our simulations the position *z* = 0 of each pore channel was set at the NPA region (see NPA and ar/R regions in Figure 2). More information about the structure of AQP1 can be found in [21, 37, 42, 43].

### 2.1. Preparation of the Model Systems

To create the NAT model system, we applied the CHARMM-GUI web server [44, 45], where the orientation of the AQP1 tetramer into the surrounding PLB was determined by the OPM database [46]. To construct the OXL model system, we used the PACKMOL package [47]. First, AQP and the POPC lipids were packed together and then 50% of the POPC molecules were replaced by POPC-ALD lipids. Finally, to build the OXP model system, we modified Cys_191_ in each monomer of the NAT system to Cys-SOH (Figures 1(a) and 1(c)) using the web server Viena-PTM 2.0 [48]. A detailed explanation of the reason for choosing a degree of oxidation of 50% in OXL and Cys_191_ as the oxidized amino acid in OXP is given in the Supplementary Information. Briefly, we used 50% oxidation in OXL, as it is large enough to observe the effect of oxidation. Moreover, we selected Cys_191_ for oxidation in OXP, since it can easily be oxidized and stays close to the ar/R selectivity region.

All model systems were energy minimized using the steepest descent algorithm. Subsequently, they were equilibrated for 150 ns in the isothermal-isobaric (NPT) ensemble at 310 K and 1 atm, applying the Nose-Hoover thermostat [49] and the semi-isotropic Parrinello-Rahman barostat [50]. We verified that this equilibration time was sufficient, as the systems reached stability after ~60 ns (cf. Supplementary Fig.  S1a). In all systems, Cl^−^ counter ions were added to the water phase to keep the systems electrically neutral. Periodic boundary conditions were applied in all Cartesian directions. The last 50 ns of the equilibration time was used to compute the average properties and to extract the starting configurations for umbrella sampling (US) simulations (see below). The MD trajectories were recorded at intervals of 20 ps. Both the equilibration and US simulations were carried out with a time step of 2 fs.

We used the GROMOS 54A7 force field parameters [51] for the interatomic interactions, in combination with the SPC water model [52]. Moreover, the GROMOS-type parameters for the RONS, the aldehyde products of the oxidized POPC (i.e., POPC-ALD), and the oxidized form of Cys (i.e., Cys-SOH) were obtained from [53–55], [56, 57], respectively. In all simulations, we used a cut-off of 1.1 nm for both the electrostatic and van der Waals interactions. We selected this cut-off after performing a series of test runs and checking that it led to stable protein structures (cf. convergence of the atomic root mean square displacements in Supplementary Fig.  S1a) and a PLB with area per lipid and thickness compatible to reference experimental data [58]. Moreover, using this cut-off, we obtained a very similar FEP of H_2_O_2_ across native AQP1 to our previously obtained FEP [32], which was not the case with other cut-off radii. All simulations and analyses of the results were performed with the GROMACS 5.1.2 package [59], except for the calculation of the pore profiles (see Section 2.2). The illustrations of the simulated systems were prepared with the VMD software [60].

### 2.2. Calculation of the Pore Profiles

We used the HOLE program [61] to calculate the pore profiles across AQP1. We calculated the average radius of the cavity of each model system along the *z*-direction, i.e., the direction passing through the pore channels (cf. Figures 1(a) and 1(b)). The program proceeds along the planes perpendicular to the direction of the channel vector (i.e., *z*-direction) and finds the largest sphere in each of these planes without overlapping with the van der Waals surface of any atom. Using the AMBER van der Waals atomic radii, we computed the final pore profile of each model system (see Supplementary Fig.  S1b) by averaging over 250 individual pore profiles, obtained from the last 50 ns of the equilibration runs.

### 2.3. Calculation of the Free Energy Profiles (FEPs)

We used the US method [62] to calculate the FEPs of H_2_O_2_, OH, NO_2_, and NO across the AQP channels of the three model systems, i.e., NAT, OXL, and OXP. To obtain the average FEP for each permeant and each model system, we used six starting configurations, which were derived from the last 50 ns of equilibration runs (i.e., at 0, 10, 20, 30, 40, and 50 ns). In each model system, we defined 72 umbrella windows along the *z*-axis at intervals of 0.1 nm. In this manner, the sampling windows spanned the whole channel ranging from the extracellular to the cytoplasmic aqueous regions (see Figures 1(b) and 2). The solutes were then inserted at the umbrella centers, and the restraining potentials were applied between the center of mass of the solutes and the alpha carbons of the NPA regions. The solutes were restrained to move along the *z*-axis by applying a harmonic bias with a force constant of 2000 kJ·mol^−1^·nm^−2^. Moreover, their lateral motion in the *xy*-plane was also restrained by using the so-called flat-bottomed position restraint, with a radius of 0.5 nm and a force constant of 500 kJ·mol^−1^·nm^−2^. The latter allowed us to insert four solutes in each plane (or umbrella window), each of which corresponding to one pore (cf. Figure 1(a)). During each US simulation, six umbrella windows, separated by 1.2 nm, were sampled simultaneously. Thus, in each US simulation, we were able to obtain the results for 24 solutes (e.g., 24 H_2_O_2_ molecules), distributed over six layers along the *z*-axis (i.e., four solutes in each plane). This procedure was done to save computation time and resources and to obtain sufficient statistics. Note that the distances between the four solutes, inserted in each plane, were greater than the cut-off, so that they did not interact with each other during the US simulation. As mentioned above, 72 umbrella windows were used or 12 US simulations were performed to obtain four FEPs, each corresponding to a single pore. Thus, the final FEP of each solute was obtained by averaging over 24 energy profiles (i.e., 4 FEPs × 6 structures). Similarly, the statistical uncertainties were obtained by calculating the standard deviation between 24 independently built FEPs. In total, 12 US × 6 structures × 4 solutes × 3 model systems = 864 US simulations were performed to obtain the FEPs. Overall, more than 1000 US simulations were carried out, if we include the test runs performed to select the most appropriate cut-off radius (see above). The US simulations were also carried out in the NPT ensemble, with the same parameters as used in the equilibration simulations. The total US simulation time lasted for 6 ns, of which the last 1 ns was used to collect the US histograms. All FEPs were constructed using a periodic version of the weighted histogram analysis method (WHAM) [63], by utilizing the *gmx wham* tool of GROMACS.

## 3. Results and Discussion

As mentioned above, we investigate the permeation of both hydrophilic (H_2_O_2_ and OH) and hydrophobic (NO_2_ and NO) RONS through the AQP1 pores of the native (NAT) and oxidized (OXL and OXP) systems. The results of the root mean square displacement (RMSD) of the alpha carbons of AQP1 (see Supplementary Fig.  S1a) show that the RMSD in all systems converges after ~60 ns. The equilibrium value fluctuates around 0.32 nm in the case of NAT and 0.33 nm in the cases of OXL and OXP, respectively. This slight increase in RMSD indicates that the oxidized systems become slightly more flexible. This can also be deduced from the average pore profile across the AQP1 channels (see Supplementary Fig.  S1b), as the fluctuations of the average pore radii in the oxidized systems (OXL and OXP) are slightly higher than in the native (NAT) system. The flexibility in AQP1, and thereby the higher fluctuations in its pore radii along the *z*-direction, may affect the permeation properties of RONS across AQP1, which is indeed observed in our simulations (see Figure 2). We observe that the minimum pore radius is invariably located at the ar/R constriction region. The obtained pore radii are 0.74 ± 0.06, 0.89 ± 0.17, and 0.85 ± 0.10 Å for the NAT, OXL, and OXP systems, respectively. This indicates again that the amino acid residues located in the pores have slightly higher fluctuations after oxidation and can possibly affect the selectivity of the constriction region.

The FEPs of the hydrophilic (H_2_O_2_ and OH) and hydrophobic (NO_2_ and NO) RONS across AQP1 of the native (NAT) and oxidized (OXL and OXP) systems are shown in Figure 2. We applied a trapezoidal correction to the FEPs, which enables to compare them with the profiles obtained for the PLB. Information about the trapezoidal correction is given in [31].

As a general trend, all permeants experience barriers when they move from the extracellular aqueous phase to the pore interior. The extent by which oxidation of the lipids (OXL) and the protein (OXP) affects the shape of the FEPs and the barrier heights depends on the permeant. In the case of H_2_O_2_, free energy barriers of 10.6 ± 2.2 kJ/mol, 13.9 ± 1.9 kJ/mol, and 14.0 ± 2.7 kJ/mol are recorded for NAT, OXL, and OXP, respectively. Oxidation appears to induce a slight increase in the free energy barrier, but the differences are still comparable to the uncertainty limits. In the case of NO_2_, we also observe a slight increase in the barrier for OXP, but not for OXL. The calculated free energy barriers are 8.9 ± 1.7 kJ/mol, 8.0 ± 1.9 kJ/mol, and 12.7 ± 1.7 kJ/mol for NAT, OXL, and OXP, respectively. Moreover, the local minimum around -0.6 nm disappears when oxidation takes place. For OH and NO, we find either a negligible (OH) or no (NO) effect of oxidation on the energy barrier, but the shape of the FEPs (i.e., local maxima and minima) is affected, as clearly seen for OXL. For OH, the free energy barriers are 7.7 ± 2.0 kJ/mol, 6.0 ± 1.9 kJ/mol, and 8.6 ± 1.8 kJ/mol for NAT, OXL, and OXP, respectively, while for NO, these values are 7.4 ± 1.4 kJ/mol, 5.5 ± 1.2 kJ/mol, and 7.2 ± 1.6 kJ/mol for NAT, OXL, and OXP, respectively. Hence, the free energy barriers are somewhat higher for H_2_O_2_ and NO_2_ than for OH and NO, which we attribute to the sizes of the species. Important to note is that the FEPs of the hydrophilic RONS (H_2_O_2_ and OH) show similarity, i.e., their local minima and maxima are located at around the same *z* positions (most obviously in the NAT case), and the same applies to the FEPs of the hydrophobic RONS (NO_2_ and NO). We should mention here that due to the high reactivity of the OH radicals, the FEPs obtained from classical nonreactive MD simulations can only provide a partial picture of their behavior in AQP, as these simulations do not account for chemical reactions. Nevertheless, it may still be helpful to know the classical free energy barriers that OH radicals need to overcome in order to have access to the pore interior. A more detailed discussion about the importance of the FEPs of OH is given in the Supplementary Information.

**Figure 3:**
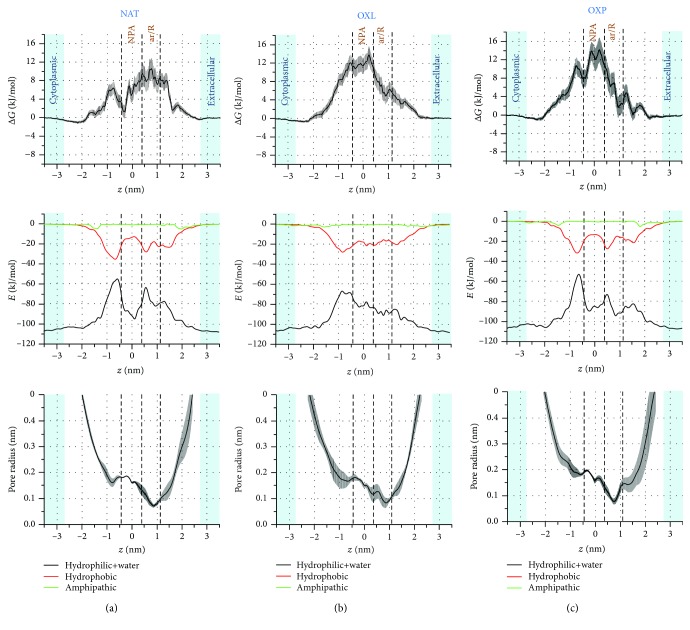
FEPs (first row) of H_2_O_2_ across the native (NAT (a)) and oxidized model systems (OXL (b) and OXP (c)), together with the nonbonded (i.e., Coulomb+van der Waals) interaction energy (second row) and pore radius (third row) profiles. The nonbonded interaction energies between H_2_O_2_ and the hydrophilic residues+water, the hydrophobic, and the amphipathic residues are shown in black, red, and green, respectively.

**Figure 4:**
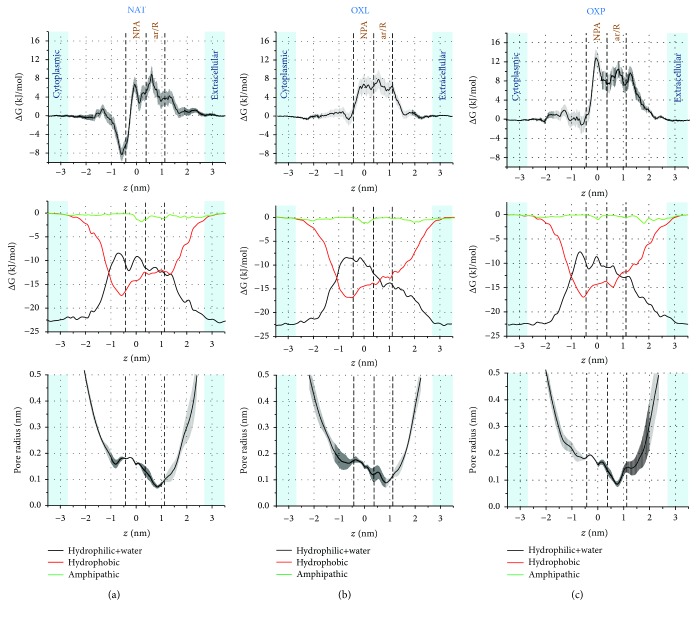
FEPs (first row) of NO_2_ across the native (NAT (a)) and oxidized model systems (OXL (b) and OXP (c)), together with the nonbonded (i.e., Coulomb+van der Waals) interaction energy (second row) and pore radius (third row) profiles. The nonbonded interaction energies between NO_2_ and the hydrophilic residues+water, the hydrophobic, and the amphipathic residues are shown in black, red, and green, respectively.

We propose a qualitative explanation for the FEPs by focusing on the nonbonded (i.e., Coulomb+van der Waals) interaction energies and pore radius profiles, as illustrated in Figures 3 and 4 for H_2_O_2_ (hydrophilic) and NO_2_ (hydrophobic), respectively. The same analysis is done for the OH and NO radicals and presented in Supplementary Information (Figs.  S2 and S3).

The nonbonded interaction energies (NBEs) along the *z*-axis are calculated between H_2_O_2_ and the hydrophilic, hydrophobic, and amphipathic amino acid residues of AQP1, as well as the water molecules located inside and outside of the AQP1 pores. To facilitate the explanation, we combine the NBE profiles for the hydrophilic residues with water (i.e., hydrophilic+water).

In all cases (i.e., NAT, OXL, and OXP), the NBE of H_2_O_2_ with the hydrophilic residues+water is higher (i.e., more negative) than with the hydrophobic and amphipathic residues. This indicates that the hydrophilic residues+water have a larger contribution for the interactions. Indeed, H_2_O_2_, being itself a hydrophilic species, strongly interacts with the hydrophilic residues and especially with water, causing the most negative energy values to be found in the extracellular and cytoplasmic aqueous regions. Furthermore, in all cases, the NBEs of H_2_O_2_ with the amphipathic residues are close to zero.

In the NAT system (Figure 3(a)), the NBE of H_2_O_2_ with the hydrophilic residues+water decreases (i.e., becomes less negative) when H_2_O_2_ penetrates from the extracellular region to the pore interior, leading to an increase in the free energy barrier (first row of Figure 3). This is only partially compensated by the increase of the NBE with the hydrophobic residues, resulting in an overall weakening of the interaction energy, which might in turn explain the increase of the free energy. The confinement experienced by H_2_O_2_ inside the channel, especially in the ar/R constriction region, creates an entropic penalty that reflects on the free energy value. The maximum free energy barrier is observed in the ar/R region, which corresponds to the minimum pore radius (third row in Figure 3). Close to the end of the ar/R region (*z* ≈ 0–0.5 nm), the NBE of H_2_O_2_ with the hydrophilic residues+water clearly increases (i.e., becomes more negative), whereas it decreases for the hydrophobic residues, resulting in an overall drop of the free energy in the NPA region. This is also attributed to a somewhat larger pore radius in the NPA region, so the interaction of H_2_O_2_ with the water molecules in the pore becomes stronger and the entropic penalty lower. Beyond the NPA region (*z*≈−0.9 nm), the pore radius slightly drops and the NBE of H_2_O_2_ with the hydrophilic residues+water becomes again weaker, while the NBE with the hydrophobic residues becomes stronger, again leading to an increase in the free energy. The changes in the NBEs are more pronounced here than in the ar/R region, showing stronger interaction of H_2_O_2_ with the hydrophobic residues and weaker interaction with the hydrophilic residues+water. Therefore, the barrier increase at *z*≈−0.9 nm is not as high as in the ar/R region.

Similar interpretations of the FEPs can be made for OXL (Figure 3(b)) and OXP (Figure 3(c)). The only difference is that the pore radii in the OXL and OXP systems are somewhat larger in the ar/R constriction region compared to that in the NAT system (see above) and have higher fluctuations along the channel. This in turn affects the NBE of H_2_O_2_ (or other permeants in general), thereby influencing the FEP. In the OXL case (Figure 3(b)), the NBE of H_2_O_2_ with the hydrophilic residues+water decreases more or less linearly (i.e., becomes less negative) from the extracellular aqueous phase till *z*≈−0.9 nm, while the NBE with the hydrophobic residues increases (i.e., becomes more negative) up to this position, being more or less constant in the ar/R and NPA regions. The drop in the NBE with the hydrophilic residues+water and the confinement lead to the free energy barrier, which reaches its maximum in the NPA region at around *z* = 0.0 nm. Beyond this position, the NBE with the hydrophobic residues rises (becomes more negative), and therefore, the free energy starts to decrease. From around *z* = −1.0 nm till the cytoplasmic region, the NBE with the hydrophilic residues+water becomes very strong, resulting in a drop in the free energy. Likewise, the FEP of the OXP case (Figure 3(c)) can also be explained from the NBEs of H_2_O_2_ with the hydrophilic residues+water and with the hydrophobic residues, just like in the NAT case.

It is interesting to note that the oxidation of Cys_191_ to sulfenic acid near the ar/R constriction turns this region more hydrophilic. As expected, the hydrophilic interactions of H_2_O_2_ in this region become stronger and the free energy value decreases. The free energy barrier, which was originally located at the ar/R constriction in the NAT system, is shifted to the other side of the channel, beyond the NPA region, in OXP. We speculate that the sulfenic acid group may affect the delicate interaction balance that maintains the bipolar orientation of the water molecules inside the channel (cf. [31]). A less organized water file inside the channel can possibly explain why the oxidation of Cys_191_ is able to affect the free energy in distal regions inside the channel. Although a similar increase of the free energy barrier was observed in OXP and OXL, the mechanisms are probably different. Both the free energy and NBE profiles are much less structured in OXL as compared to OXP. It is well known that lipid oxidation decreases the membrane thickness [64–66], which in turn has an impact on the AQP properties [67]. We hypothesize that the change in membrane thickness may disturb the AQP structure and impact the water file organization in a similar fashion as in the case of Cys_191_ oxidation. It is tempting to draw a parallel between the increase in the free energy barrier witnessed upon oxidation and the alleged oxidative gating of AQPs [33–35].

We do not provide a detailed explanation for the FEP of the hydrophobic NO_2_ (see Figure 4), as the analysis is very similar as for H_2_O_2_. We concentrate mainly on the difference in the NBE profiles of NO_2_ and H_2_O_2_, because they help to explain the observed FEPs. As is clear from Figure 4, the strongest NBE is observed again in the extracellular and cytoplasmic aqueous regions (i.e., around -23 kJ/mol, see black curve in the second row), which corresponds to the interaction between NO_2_ and water. Note that this energy is ~5 times lower than the energy obtained for H_2_O_2_ in these regions, due to the hydrophobicity of NO_2_.

On the other hand, the maximum NBE with the hydrophobic residues is around -17.5 kJ/mol (see red curve in second row of Figure 4), which is only ~2 times lower than the NBE of H_2_O_2_ with the hydrophobic residues (cf. Figure 3). This indicates that although the NBE of H_2_O_2_ is stronger with both the hydrophilic residues+water and the hydrophobic residues, compared to the NBE of NO_2_, the interaction of NO_2_ with the hydrophobic residues is relatively stronger, as expected based on its hydrophobicity. Indeed, the deep minimum of the FEP observed in the NAT case (i.e., at around *z* = −0.6 nm, see Figure 4(a)) is due to the stronger interaction of NO_2_ with the hydrophobic residues compared to the interaction with the hydrophilic residues+water. This minimum in the FEP disappears in the case of OXL and OXP (Figures 4(b) and 4(c)), most likely due to the higher fluctuations in the pore radius profiles. The other characteristics of the FEPs can be explained as was done above for H_2_O_2_. A similar analysis can also be made for the FEPs of the OH and NO radicals (see Figs.  S2 and S3).

Thus, in general, the calculated NBE and pore radius profiles help to explain the influence of the different types of amino acid residues and of water molecules on the FEPs of RONS. The NBEs of the hydrophilic RONS are much stronger than those of the hydrophobic species (cf. Figures 3 and 4, as well as Figs.  S2 and S3). For the hydrophilic species (H_2_O_2_ and OH), the NBE contributions of the hydrophilic residues+water to the overall interactions are higher than the contributions of the hydrophobic residues. Analogously, for the hydrophobic species (NO_2_ and NO), the NBE contributions of the hydrophobic residues to the overall energy are higher than the contributions of the hydrophilic residues+water.

It should be mentioned that the central cavity of the AQP tetramer (see Figure 1(a)) may also play a role in the transport of RONS into the cell interior. However, we do not consider the contribution of the central cavity because our previous studies showed that the obtained free energy barriers of the hydrophilic species (OH and H_2_O_2_) through the central cavity are much higher than through individual pores [31, 32]. These barriers are either similar or higher than those calculated at the native PLB. Thus, it is highly conceivable that the hydrophilic species most probably permeate across the pores, even when lipid or protein oxidation takes place, as they experience lower free energy barriers. The hydrophobic species (NO and NO_2_), on the other hand, most likely prefer the transport across the PLB, as they experience significantly low permeation barriers (i.e., ~1 kJ/mol) [55]. These barriers are negligible compared to those through AQP pores obtained in this study using both lipid and protein oxidation.

Finally, it should also be mentioned that real cell membranes are vastly more complex than the simple membrane models considered here. Cell membranes are made of a complex mixture of lipids and embedded membrane proteins, which are able to segregate and form heterogeneous domains at the nanometer length scale. The expression of membrane proteins such as AQPs is subject to intricate regulation mechanisms which are also expected to play a role in the cellular response to oxidative stress. With that in mind, the effects of oxidative stress on AQP permeability, as unveiled by our simulations, should be viewed as part of a larger and more complex biochemical machinery.

## 4. Conclusions

The aim of our study was to better understand the permeation process of both hydrophilic (H_2_O_2_ and OH) and hydrophobic (NO_2_ and NO) RONS across the pores of AQP1, which is a transmembrane protein, as well as the effect of both lipid and protein oxidation, which can be induced by these RONS.

Our results showed that oxidation of a single Cys_191_ residue in each pore of AQP1 (i.e., protein oxidation) has almost the same effect on the free energy barrier of H_2_O_2_ as 50% lipid oxidation in the PLB. For OH and NO, the barrier in case of protein oxidation is even slightly higher, while it is clearly higher in the case of NO_2_ compared to 50% lipid oxidation. Overall, both lipid and protein oxidation influences the shape of the FEPs of all RONS, as well as the barrier heights for H_2_O_2_ and NO_2_. In general, the free energy barriers are somewhat higher for H_2_O_2_ and NO_2_ than for OH and NO, which we attributed to the sizes of these species.

To explain the FEPs, we studied the NBEs of the RONS with the hydrophilic and hydrophobic amino acid residues and water, found both inside and outside of the pores, as well as the pore radius profiles. They help to explain the shape of the FEPs, by showing the amino acid residues and water molecules involved in the permeation process.

Our simulation results indicate that, overall, oxidation does not strongly affect the transport of RONS through AQP. However, we note that regular MD simulations are unable to capture large-scale protein structural changes that happen beyond the multimicrosecond time domain. As the barrier for transport of hydrophilic RONS through AQP (both native and oxidized) is quite low (~6-12 kJ/mol), we suggest that these species (i.e., OH and especially H_2_O_2_) will enter the cell through AQPs, as they experience a clearly higher barrier through the PLB (~15-30 kJ/mol) [32, 53, 65], especially in the native (i.e., unstirred) lipid bilayer. On the other hand, the hydrophobic RONS (i.e., NO, NO_2_, but also others, like O_2_) most likely permeate through the PLB, where lipid (per)oxidation can take place, as their barrier through AQP is higher (i.e., ~5-12 kJ/mol vs. ~1 kJ/mol for the PLB [55]).

Our results provide molecular level insight into the processes of RONS permeation through the membrane and specifically into the role of AQP, which is often more expressed in cancer cells. This may help to improve our understanding on the selective rise of RONS observed in cancer cells, shedding light on the selective anticancer mechanism of CAP.

## Data Availability

The data used to support the findings of this study are available from the corresponding author upon request.

## Supplementary Materials

Supplementary Materials Supplementary Information: further details about the chosen oxidized model systems and the three supplementary figures.
