# Supplementary material for: Transport of Reactive Oxygen and Nitrogen Species across Aquaporin: A Molecular Level Picture
Source: Oxid Med Cell Longev. 2019 Jun 17;2019:2930504. doi: 10.1155/2019/2930504 (PMC6604302; doi:10.1155/2019/2930504)
Supplement: Supplementary Materials — Supplementary Information: further details about the chosen oxidized model systems and the three supplementary figures. [file 2930504.f1.pdf]

Supplementary Information

Transport of reactive oxygen and nitrogen species across aquaporin: a molecular level picture

Maksudbek Yusupov[1], Jamoliddin Razzokov[1], Rodrigo M. Cordeiro[2] and Annemie Bogaerts[1]

[1] Research group PLASMANT, Department of Chemistry, University of Antwerp, Universiteitsplein 1, B-2610 Antwerp, Belgium
[2] Centro de Ciências Naturais e Humanas, Universidade Federal do ABC, Avenida dos Estados 5001, CEP 09210-580 Santo André, SP, Brazil

E-mail: maksudbek.yusupov@uantwerpen.be

OXL and OXP model systems

In the OXL system (i.e., the system with oxidized lipid bilayer) we use 50% oxidation, which is large enough to observe the effect of oxidation, but not too large to cause direct pore formation. We create the OXL system such that the oxidized lipid molecules (i.e., POPC-ALD, see Fig. 1d in the main paper) are homogeneously distributed throughout the lipid system surrounding AQP1 in both leaflets (cf. Fig. 1b in the main paper). This is done, since our test simulations with randomly created oxidized lipid systems showed that if the oxidized lipids are created in close vicinity, this may cause pore formation in the phospholipid bilayer (PLB). In other words, if the oxidized lipids are created adjacently, the oxidation degree in a local area of the bilayer may reach 100%, which indeed results in pore formation [1]. We are sure that within the simulation time applied in this study (i.e., 150 ns equilibration and 6 ns umbrella sampling (US) runs afterwards) we do not observe any lipid pore formation. We presume that the lipid pore formation may still occur at longer times, when the oxidized lipids gather in one area of the PLB through lateral diffusion, forming locally 100% of oxidation. However, this is beyond the time of our simulations and therefore we do not observe this effect with homogeneously distributed oxidized lipids.

In the OXP system (i.e., the system with oxidized protein) we modify the Cys_{191} residues of each pore channel in AQP1 to Cys sulfenic acid (i.e., Cys-SOH, see Fig. 1a, c in the main paper). The reasons for choosing Cys_{191} are the following. Cys, among other highly reactive amino acids (Trp, Tyr and Met, see Fig. 1c in the main paper), has the highest rate constant for reaction with OH radicals [2]. Secondly, Cys_{191} is located in the pore (namely, in the ar/R region), which gives the OH radicals access to react. Indeed, OH radicals have a local minimum in the ar/R region (see the free energy profile (FEP) of OH in the NAT and OXL cases, in Fig. S2 below), which most probably leads to oxidation of this residue. Note that the diffusion of OH radicals is limited due to their high reactivity; these radicals may react with other compounds before reaching the Cys_{191} residues, although the latter are located closer to the extracellular aqueous region. On the other hand, our previous studies with reactive molecular dynamics (MD) simulations revealed that the transport of OH radicals in water is not only governed by diffusion, but also through H-abstraction reaction with water molecules [3, 4]. Indeed, OH radicals may react with water, abstracting a hydrogen atom and forming a new water molecule and OH radical. This process can continuously repeat during the transport of OH radicals into AQP pores. Hence, it is conceivable that some of the OH radicals may be able to reach the vestibular Cys_{191} residues and oxidize them. It may also be possible that a few of them can even transport further through the pores and reach the cell interior, since their free energy barriers are relatively low (see Fig. S2 below). In this sense, the FEPs of OH radicals across AQP1, obtained by our classical non-

reactive MD simulations, may be important in order to know how high is the barrier they can experience if they do not react with the residues during the transport through the pores. It is important to note that in a recent study using AQP8 [5], the authors found that H_2S can block H_2O_2 transport across AQP8 pores. They proposed that the sulfenylation (i.e., Cys-SOH formation) most probably takes place on Cys_{53}, located in the ar/R constriction region (similar to Cys_{191} in AQP1), which subsequently results in the persulfidation of these residues (i.e., Cys-SSH formation) by H_2S, thereby preventing the transport of H_2O_2 [5]. They also stated that the sulfenylation itself is not sufficient to block the transport through the AQP8 channel, but this (prior) oxidation of Cys_{53} is required for persulfidation by H_2S. We also observed that the sulfenylation does not lead to a complete inhibition of the RONS permeation, although a slight increase in free energy barrier is obtained (see Fig. 2 in the main paper).

Results

Figure S1 illustrates the time evolution of the root mean square displacement (RMSD) of the alpha carbons of AQP1, as well as the average pore profile across the AQP1 channels, for the NAT, OXL and OXP systems.

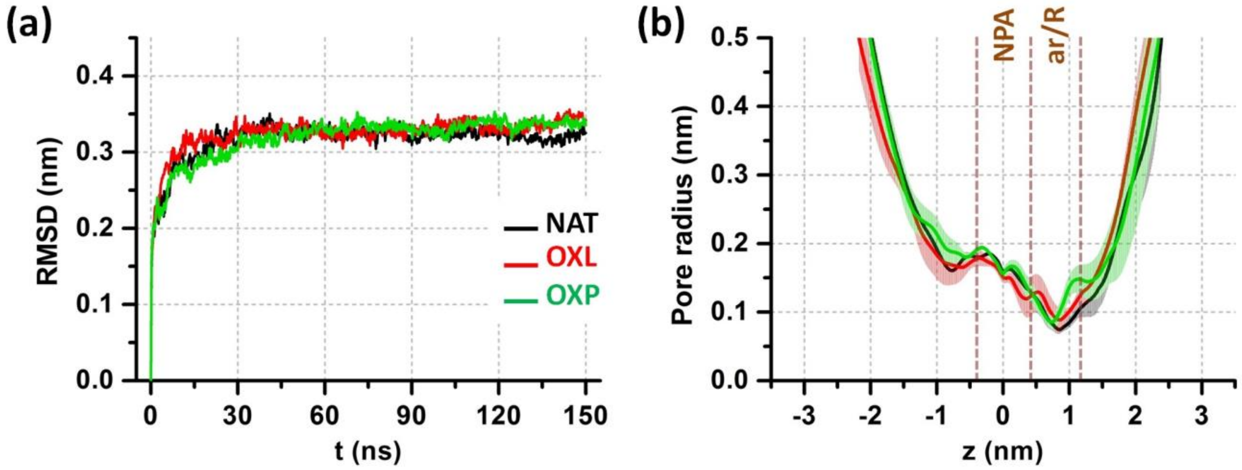

Figure S1. *RMSD of the alpha carbons of AQP1 (a) and average pore radius profile across the AQP1 channels (i.e., along the z direction) (b), of the native (NAT) and oxidized (OXL and OXP) systems. The uncertainties of the pore profiles in (b) are represented in pale color. The NPA and ar/R regions are indicated by the brown dashed lines and z=0 nm is set to the NPA region. The same designation of the NPA and ar/R regions applies to the other similar figures below.*

The first rows of Figures S2 and S3 illustrate the FEPs of OH and NO across the native (a) and oxidized (b and c) model systems. The results displayed in the second rows (i.e., the non-bonded energy profiles) and third rows (pore profiles) are used to explain these FEPs. The non-bonded energies are calculated between OH (or NO) and the hydrophilic, hydrophobic and amphipathic residues of AQP1, as well as with water located both inside and outside of the AQP1 pores. To facilitate the explanation, the non-bonded energy profiles of the hydrophilic residues and water are combined.

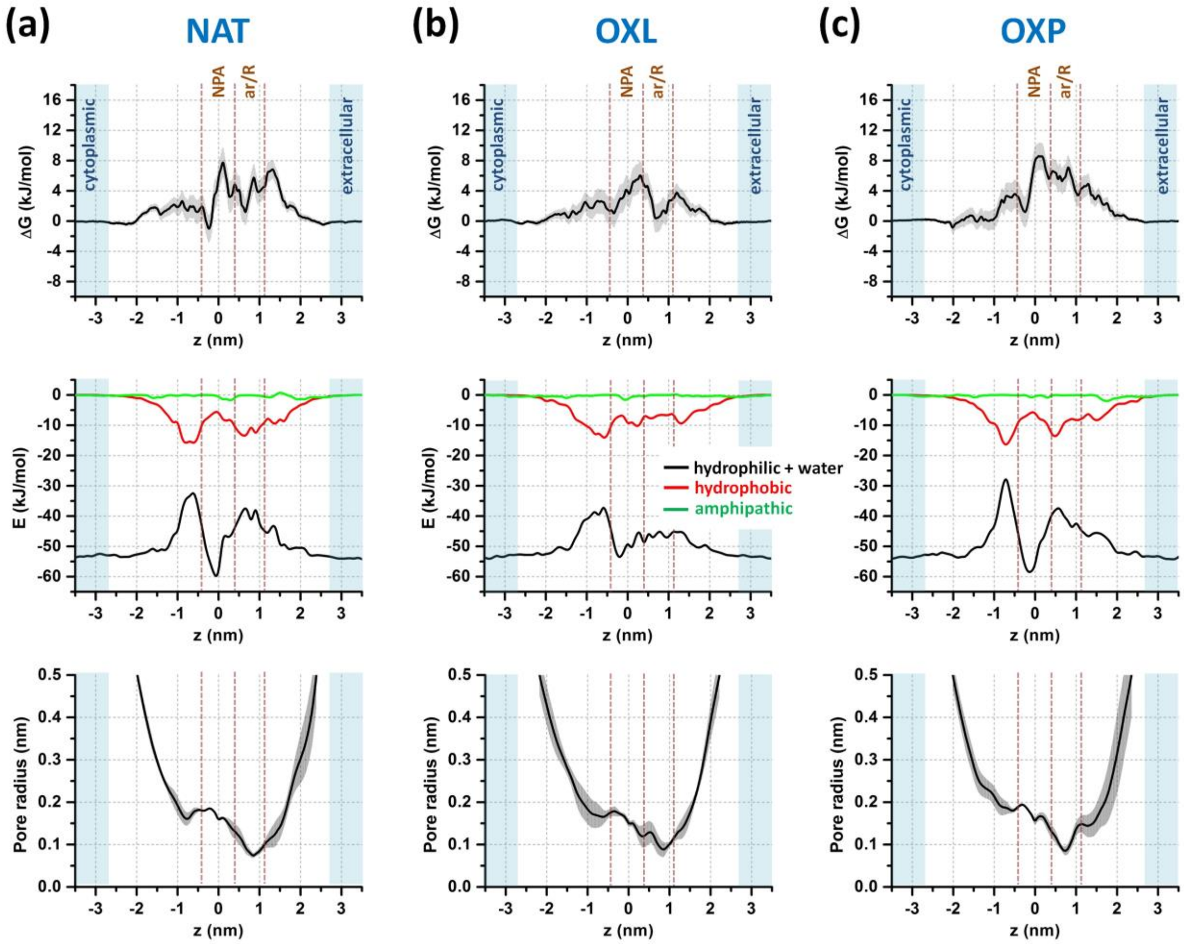

Figure S2. *FEPs (first row) of OH across the native (NAT (a)) and oxidized model systems (OXL (b) and OXP (c)), together with the non-bonded (i.e., Coulomb + van der Waals) interaction energy (second row) and pore radius (third row) profiles. The non-bonded interaction energies between OH and the hydrophilic residues + water, the hydrophobic and the amphipathic residues are shown in black, red and green, respectively. The extracellular and cytoplasmic aqueous phases are shown in light blue color.*

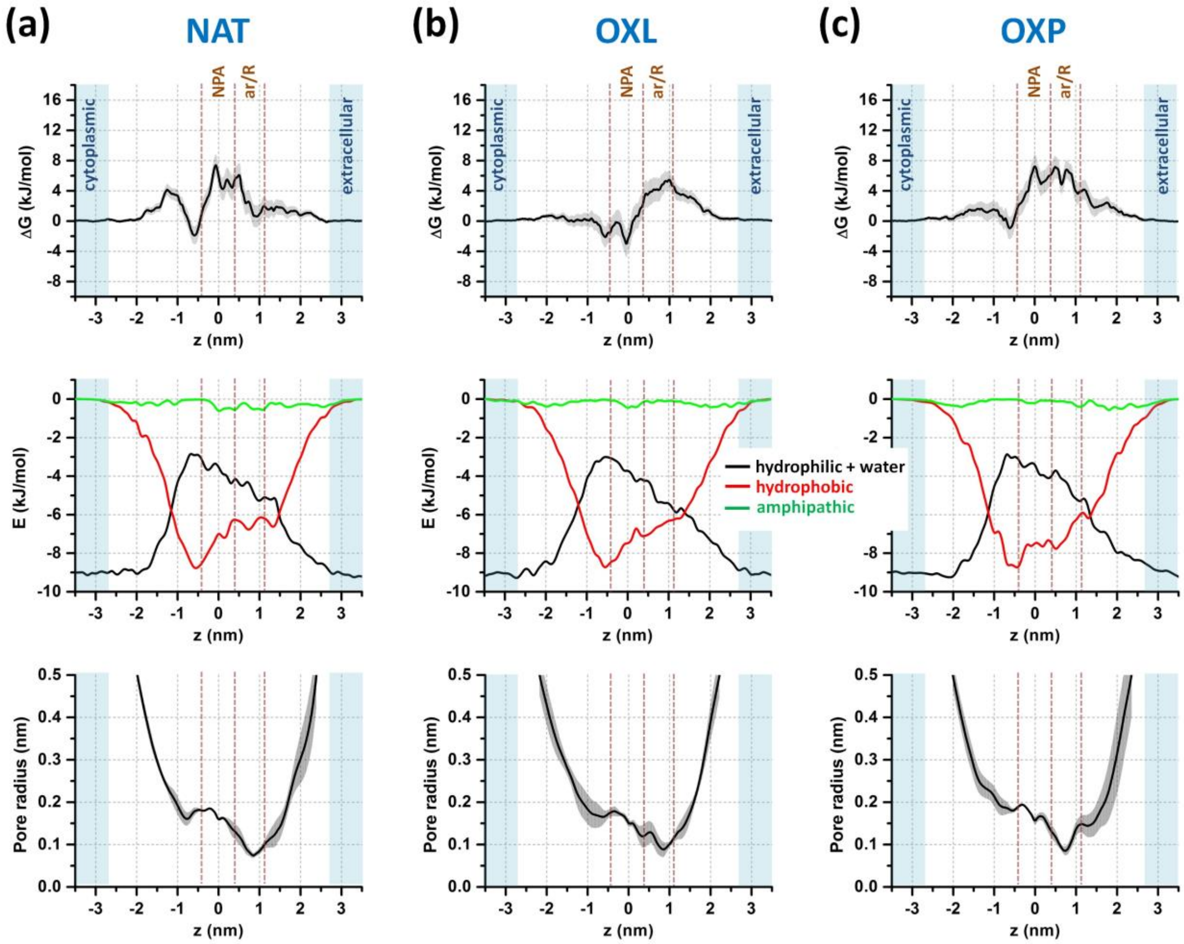

Figure S3. *FEPs (first row) of NO across the native (NAT (a)) and oxidized model systems (OXL (b) and OXP (c)), together with the non-bonded (i.e., Coulomb + van der Waals) interaction energy (second row) and pore radius (third row) profiles. The non-bonded interaction energies between NO and the hydrophilic residues + water, the hydrophobic and the amphipathic residues are shown in black, red and green, respectively. The extracellular and cytoplasmic aqueous phases are shown in light blue color.*

References

[1] Van der Paal J, Neyts E C, Verlackt C C and Bogaerts A 2016 Effect of lipid peroxidation on membrane permeability of cancer and normal cells subjected to oxidative stress *Chemical Science* **7** 489-98

[2] Xu G and Chance M R 2007 Hydroxyl Radical-Mediated Modification of Proteins as Probes for Structural Proteomics *Chemical Reviews* **107** 3514-43

[3] Yusupov M, Neyts E C, Simon P, Berdiyorov G, Snoeckx R, van Duin A C T and Bogaerts A 2013 Reactive molecular dynamics simulations of oxygen species in a liquid water layer of interest for plasma medicine *Journal of Physics D: Applied Physics* **47** 025205

[4] Verlackt C C W, Neyts E C and Bogaerts A 2017 Atomic scale behavior of oxygen-based radicals in water *Journal of Physics D: Applied Physics* **50** 11LT01

[5] Bestetti S, Medraño-Fernandez I, Galli M, Ghitti M, Bienert G P, Musco G, Orsi A, Rubartelli A and Sitia R 2018 A persulfidation-based mechanism controls aquaporin-8 conductance *Science Advances* **4** eaar5770
